# Introducing WikiPathways as a Data-Source to Support Adverse Outcome Pathways for Regulatory Risk Assessment of Chemicals and Nanomaterials

**DOI:** 10.3389/fgene.2018.00661

**Published:** 2018-12-21

**Authors:** Marvin Martens, Tim Verbruggen, Penny Nymark, Roland Grafström, Lyle D. Burgoon, Hristo Aladjov, Fernando Torres Andón, Chris T. Evelo, Egon L. Willighagen

**Affiliations:** ^1^Department of Bioinformatics – BiGCaT, NUTRIM, Maastricht University, Maastricht, Netherlands; ^2^Institute of Environmental Medicine, Karolinska Institutet, Stockholm, Sweden; ^3^Department of Toxicology, Misvik Biology, Turku, Finland; ^4^U.S. Army Engineer Research and Development Center, Vicksburg, MS, United States; ^5^Organisation for Economic Co-operation and Development Environment Directorate, Paris, France; ^6^Laboratory of Cellular Immunology, Humanitas Clinical and Research Institute, Rozzano, Italy; ^7^Center for Research in Molecular Medicine and Chronic Diseases, University of Santiago de Compostela, Santiago de Compostela, Spain; ^8^Maastricht Centre for Systems Biology, Maastricht University, Maastricht, Netherlands

**Keywords:** adverse outcome pathways, risk assessment, omics, WikiPathways, interoperability

## Abstract

A paradigm shift is taking place in risk assessment to replace animal models, reduce the number of economic resources, and refine the methodologies to test the growing number of chemicals and nanomaterials. Therefore, approaches such as transcriptomics, proteomics, and metabolomics have become valuable tools in toxicological research, and are finding their way into regulatory toxicity. One promising framework to bridge the gap between the molecular-level measurements and risk assessment is the concept of adverse outcome pathways (AOPs). These pathways comprise mechanistic knowledge and connect biological events from a molecular level toward an adverse effect outcome after exposure to a chemical. However, the implementation of omics-based approaches in the AOPs and their acceptance by the risk assessment community is still a challenge. Because the existing modules in the main repository for AOPs, the AOP Knowledge Base (AOP-KB), do not currently allow the integration of omics technologies, additional tools are required for omics-based data analysis and visualization. Here we show how WikiPathways can serve as a supportive tool to make omics data interoperable with the AOP-Wiki, part of the AOP-KB. Manual matching of key events (KEs) indicated that 67% could be linked with molecular pathways. Automatic connection through linkage of identifiers between the databases showed that only 30% of AOP-Wiki chemicals were found on WikiPathways. More loose linkage through gene names in KE and Key Event Relationships descriptions gave an overlap of 70 and 71%, respectively. This shows many opportunities to create more direct connections, for example with extended ontology annotations, improving its interoperability. This interoperability allows the needed integration of omics data linked to the molecular pathways with AOPs. A new AOP Portal on WikiPathways is presented to allow the community of AOP developers to collaborate and populate the molecular pathways that underlie the KEs of AOP-Wiki. We conclude that the integration of WikiPathways and AOP-Wiki will improve risk assessment because omics data will be linked directly to KEs and therefore allow the comprehensive understanding and description of AOPs. To make this assessment reproducible and valid, major changes are needed in both WikiPathways and AOP-Wiki.

## Introduction

The last decades have seen many developments in risk assessment strategies for an ever-growing number of chemicals and nanomaterials, aiming to reduce the use of animals and cost of risk assessment and to increase the predictive value. In parallel to these changes, experimental approaches in regular toxicology research have also made major steps setting up novel high-throughput technologies for generating large-scale (omics) datasets such as transcriptomics, metabolomics, and proteomics. However, these technologies are not consistently implemented in regulatory risk assessment and there is a need for proper integration of knowledge, testing systems, and analysis tools for these approaches to be of added value over existing methodologies in risk assessment.

To support the paradigm shift toward animal-free, cheap and more effective risk assessments of chemicals, the concept of adverse outcome pathways (AOPs) emerged (Ankley et al., 2010), which integrate mechanistic knowledge of the toxicological effects of chemical compounds and nanomaterials and thereby assist integrated approaches to testing and assessment strategies. AOPs are structured as logical sequences of causally linked and measurable biological events [key events (KEs)] that occur after exposure to a stressor that triggers a biological perturbation, called the molecular initiating event (MIE). These KEs are connected by Key Event Relationships (KERs) and describe the downstream effects on increasing levels of biological organization, from molecular, cellular, tissue, organ, individual, and population responses toward an adverse outcome (AO) (Villeneuve et al., 2014; Leist et al., 2017; Vinken et al., 2017).

The Organisation for Economic Co-operation and Development (OECD) was the first organization to embrace AOPs by launching the AOP Development Programme in 2012 for the establishment of AOPs in a qualitative way and provide guidance material for standardized, structured development of AOPs (Vinken, 2013; Organisation for Economic Co-operation and Development [OECD], 2017). With that, the AOP Knowledge Base (AOP-KB^1^) emerged in 2014 as a collective platform of various tools to assist in the development of AOPs. Its main components are the AOP-Wiki^2^, Effectopedia^3^ and the AOPXplorer Cytoscape application.

The AOP-Wiki is the result of collaboration between the European Commission’s Joint Research Center (JRC) and the United States Environmental Protection Agency (US EPA). It is developed to be a central knowledge-sharing platform which facilitates cooperative development of AOPs and strictly follows the OECD’s guidance materials for AOP development. Nowadays, it is the most actively used module of the AOP-KB and with the recent efforts on annotation with ontology tags, it has been aiming for semantic interoperability. This started with the development of the AOP Ontology (Burgoon, 2017) and recently, the addition of various other ontologies to match the various domains described in AOPs, from Gene Ontology for biology annotation toward the Population and Community Ontology for annotation of events on the population level (Ives et al., 2017).

Effectopedia (Watanabe et al., 2018) is another tool from AOP-KB, developed by OECD, dedicated to the collaborative development of quantitative AOPs. The AOP diagram is the focal point of its user interface providing visual means for adding new and navigation through existing AOP elements, offering easy access to their description. In addition to KE and KER, Effectopedia also has an explicit representation of test methods, collected data and executable models. The integration of response data in KER allows the system to predict downstream KEs using measurements or models for upstream KEs that can be measured using *in chemico*, high throughput and or *in vitro* methods. The goal of fully quantified AOPs is to allow the prediction of an adverse outcome in time and magnitude using a minimum number of experimental measurements for KE responses that cannot be adequately modeled by other means.

The third is AOPXplorer, a Cytoscape application, meant for building networks of KEs, forming AOP Networks (AOPNs) and allow data visualization of various types on top of the AOPNs. The goal of AOPXplorer is to help investigators and risk assessors understand how chemical exposures result in information flow throughout the AOPN, allowing them to make defensible stories and inferences about potential adverse outcomes.

It has been postulated that omics technologies can be used for various goals in regulatory toxicology, such as biological read-across based on molecular events to prioritize chemicals for testing, cross-species extrapolation to link to evolutionary biology and the identification of KEs (Hartung, 2016). Although omics approaches have already been used in toxicology to define specific modes of action (Edwards and Preston, 2008) or identifying biomarkers (Grafström et al., 2015), they have not found their way into regulatory acceptance for assessment of chemicals and nanomaterials (Buesen et al., 2017; van Ravenzwaay et al., 2017). There is a need for well-established experimental protocols for data generation, storage, processing, analysis, and interpretation to reach regulatory acceptance. Besides, an integration framework for data interpretation to identify relevant molecular changes and pathways is required, as well as the filling of knowledge gaps that keep risk assessors from causally linking molecular events to an adverse outcome at a higher level of biological organization (Brockmeier et al., 2017; Buesen et al., 2017; Sauer et al., 2017; Vachon et al., 2017; Campos and Colbourne, 2018). Taken together, the level of uncertainties and inconsistencies in experimental design should be minimized to allow omics approaches in risk assessment and AOPs. So far, various ideas have emerged to introduce omics data to the concept of the AOPs, such as a pipeline for KE enrichment (Nymark et al., 2018), workflow for computationally predicted AOPs from public data (Bell et al., 2016) and the Transcriptomics Reporting Framework (Gant et al., 2017).

There is a demand for a consistent, well-defined protocol to analyze and integrate the data in order to describe the molecular effects downstream of an MIE (Brockmeier et al., 2017). Molecular pathway databases and tools exist to analyze omics datasets through pathway analysis, which happens through probability scoring of pathways containing differently expressed genes and thereby reducing the number of dimensions of omics datasets to the number of biological pathways. Various molecular pathway databases exist which could be viable tools for the integration of omics approaches in regulatory risk assessment, such as KEGG (Kanehisa and Goto, 2000), Reactome (Fabregat et al., 2018) and WikiPathways (Slenter et al., 2018).

In this paper, we describe how WikiPathways ^4^ (Slenter et al., 2018) an open-science molecular pathway database which captures mechanistic knowledge in pathway diagrams, can be a supportive database for AOPs and the analysis and interpretation of omics datasets through pathway analysis. WikiPathways has similar levels of coverage of genes and metabolites as Reactome and KEGG (Kutmon et al., 2016; Slenter et al., 2018) and performs better in covering signaling pathways (Azad et al., 2017). This can be done with PathVisio (Kutmon et al., 2015), a pathway diagram drawing tool that is connected to WikiPathways, in which omics data can be visualized and pathway analysis can be performed. Also, WikiPathways exists as a Cytoscape application, which allows the same pathways to be used for network analysis (Kutmon et al., 2014).

**FIGURE 1:**
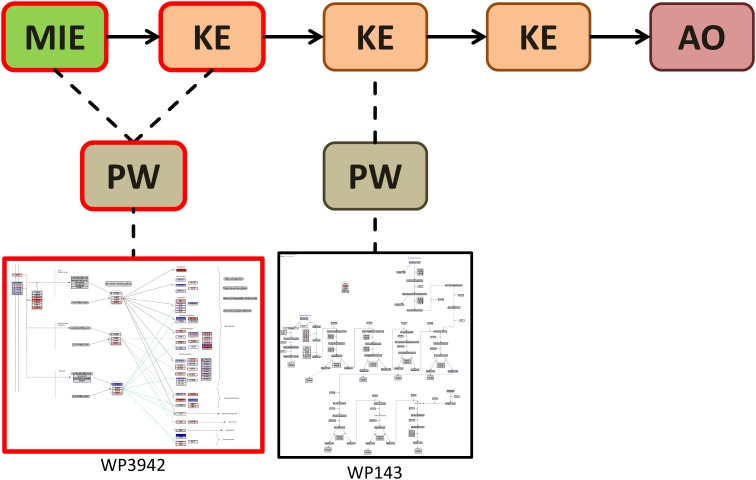
Illustrative description of the linkage of KEs of an AOP with molecular pathways described in WikiPathways and the practical application of transcriptomics. Transcriptomics and pathway enrichment analysis are commonly used to elucidate molecular pathways affected after exposure to a chemical or stress signal. In this illustration, gene expression levels in WP3942 (Adriaens et al., 2018) are significantly changed (red and blue nodes in the pathway diagram, for up- and downregulation). Because this pathway is linked to the MIE and first KE, these are hypothetically affected by the chemical, highlighted with red borders and require validation. WP143 (Hanspers and Slenter, 2017) is not affected by the exposure of this chemical at the same time and dose, and the KE that is linked to this biological pathway is not considered to be affected but could follow later or at a higher dose. AO, adverse outcome; KE, key event; MIE, molecular initiating event; PW, pathway; WP, WikiPathways.

Thanks to the adaptability and accessibility of WikiPathways, communities can collaborate on creating, assessing and improving the understanding of molecular pathways (Pico et al., 2008). Therefore, WikiPathways could be a valuable tool for the risk assessment community. It can provide improved molecular descriptions of early KEs which support biological plausibility. At the same time, it can serve as empirical support to KERs and allow the integration of omics technologies in the concept of AOPs in a systematic manner. As illustrated in Figure 1, ideally, all KEs in AOP-Wiki are linked by at least one molecular pathway, which can be highlighted by omics analysis and thereby revealing KEs. However, WikiPathways needs to be integrated with the existing modules in the AOP-KB. Here, we focus on the AOP-Wiki by describing its current implementation of semantic annotations and we will show how we can connect the AOP-Wiki with WikiPathways through identifiers for genes, proteins and metabolites, and ontologies (Bard and Rhee, 2004), which are pre-defined vocabularies used to describe knowledge and assist in the integration of data sources. Furthermore, we will propose a strategy for future work on connecting the two databases, describing the planned work on WikiPathways and suggestions for improving the AOP-Wiki and its contents to allow linkage of databases.

## Materials and Methods

### Retrieval of AOP-Wiki Data

The AOP-Wiki allows the use of their data for publication purposes, by storing permanent quarterly downloads on the website^5^. For this paper, we used the AOP-Wiki XML file of April 1st, 2018, containing all AOP-Wiki content.

### Parsing the AOP-Wiki XML

The AOP-Wiki XML was parsed with Python 3.5 (Python Software Foundation, 2010) and the ElementTree XML API with the “.parse”-function which resulted in an ElementTree wrapper class that represents an entire element hierarchy. The information, that was required for the experiments, was extracted included stressor information, ontology annotations, and information on KEs and KERs. The source code, as well as a brief tutorial on the execution of it, are available on GitHub (Martens, 2018).

### BridgeDb Identifier Mapping in R

In order to perform identifier mapping for the chemicals that are stored on AOP-Wiki with CAS Registration Numbers (CAS numbers), we used the BridgeDb, an identifier mapping framework (Van Iersel et al., 2010). The CAS numbers from the AOP-Wiki were saved as plain text file and imported in RStudio (version 1.1.447; R version 3.4.4) (R Core Team, 2013; R Studio Team, 2015), in which the R-package BridgeDbR (Leemans et al., 2018) was utilized to map the CAS numbers to ChEBI identifiers with the BridgeDb metabolite identifier mapping dataset (Slenter, 2018). The R code used for the identifier mapping is available on GitHub along with a tutorial to execute the script (Martens, 2018).

### WikiPathways Data

Information from WikiPathways was retrieved using the WikiPathways SPARQL endpoint^6^ (Waagmeester et al., 2016), version 20180610. SPARQL is a query language to select specific subsets of data from a collection of RDF, a standard framework for knowledge descriptions. For this manuscript, various queries were performed to request information about WikiPathways’ use of ontologies and to retrieve pathways for lists of genes related to KEs.

### Textual Identifier Mapping for Genes and Proteins

In order to perform identifier mapping on the free-text descriptions of AOP-Wiki, we downloaded a human gene identifier dataset from the HUGO Gene Nomenclature Committee (HGNC) (HUGO Gene Nomenclature Committee [HGNC], 2018) in May 2018 via genenames.org, a curated online repository for HGNC-approved gene nomenclature, gene families and associated resources (Yates et al., 2017). A custom download was performed in which we requested HGNC IDs, approved symbols, approved names, previous symbols, synonyms, and Ensembl IDs. These identifiers were loaded in Python and used to filter the descriptions of KEs for genes, which are filtered for KEs on the molecular, cellular, tissue, and organ level of biological organization. Also, the KERs that connect these KEs were parsed and identifiers were mapped on their descriptions and texts on biological plausibility and empirical support.

### Manual Matching of AOP-Wiki KEs to Molecular Pathways on WikiPathways

All AOP-Wiki KE IDs on the molecular, cellular, tissue, and organ level were extracted and their corresponding web pages were opened on aopwiki.org. From the KE titles and descriptive text, pathway names were selected and queried on wikipathways.org via the search-bar for molecular pathways. If results showed up for this initial search, the KE was considered present in WikiPathways. If the KEs did not contain a direct mention of a pathway, the genes and proteins were noted and were queried for their presence in pathways via the WikiPathways SPARQL endpoint. For KEs at the cellular level, at least the majority of the genes and proteins should be present in at least one pathway. However, for molecular KEs that describe only an interaction between two molecules, only the presence of the target molecule in WikiPathways was necessary to consider the KE covered by WikiPathways. This method was meant to give a rough overview of the overlap between the AOP-Wiki and WikiPathways databases. Because it does not include synonyms or ontological similarity, this overview is expected to underestimate the overlap.

## Results

**FIGURE 2:**
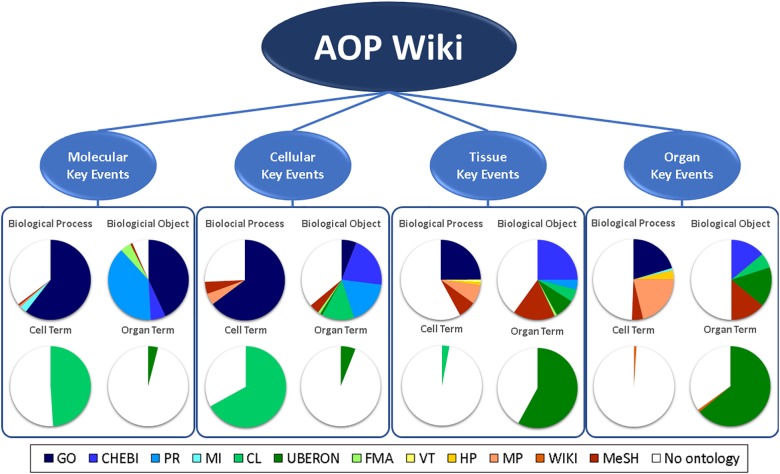
Ontology usage of AOP-Wiki for KEs on the molecular, cellular, tissue, and organ level of biological organization. GO, gene Ontology; CHEBI, chemical entities of biological interest; PRO, protein ontology; MI, molecular interactions; CL, cell ontology; UBERON, uber anatomy ontology; FMA, foundational model of anatomy; VT, vertebrate trait; HP, human phenotype ontology; MP, mammalian phenotype ontology; WIKI, AOP-Wiki; MeSH, medical subject headings.

**FIGURE 3:**
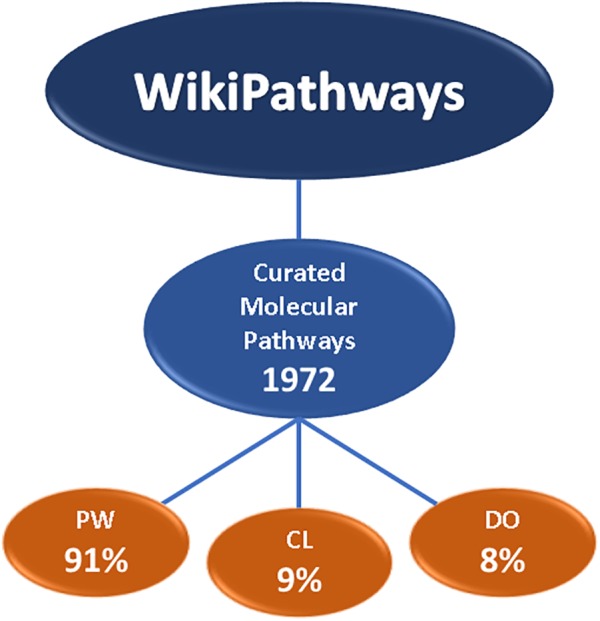
WikiPathways statistics. The total number of molecular pathways in the WikiPathways database, and the level of pathway annotations with onology tags. PW, pathway ontology; CL, cell ontology; DO, disease ontology.

For hard linkage of the two databases, meaning explicit identifier matching, we looked at the usage of ontology annotations of the AOP-Wiki and WikiPathways. For the AOP-Wiki we extracted ontology annotations from KEs on the molecular, cellular, tissue and organ level and identified which ontology sources were currently in use for biological processes, biological objects, cell-terms, and organ-terms. As shown in Figure 2, a large amount of KEs are not yet annotated with ontology tags. When looking more in detail, one can notice that biological processes are mostly described with Gene Ontology (GO) tags, especially at the molecular and cellular KEs whereas the biological objects are mostly annotated with tags from ChEBI and Protein Ontology (PR). Although AOP-Wiki contains various ontology sources, WikiPathways only uses three: Pathway Ontology (PW), Cell Ontology (CL), and the Disease Ontology (DO) (Figure 3). However, apart from the CL for a contextual description of the process, WikiPathways and AOP-Wiki do not share ontologies for other biological elements.

Although no direct mappings through ontologies are possible at the moment of writing this paper, an alternative approach for hard linkage is the mapping of chemicals, metabolites, and genes to WikiPathways. Although we do not expect to find many of the AOP-Wiki stressor chemicals in WikiPathways, we wanted to identify the existing overlap of chemicals between the two databases nevertheless. First, we found all 306 stressors, describing 207 chemicals, which were annotated with 205 CAS numbers. We mapped these CAS numbers to ChEBI IDs in R with BridgeDbR and created a SPARQL query to find all pathways that have any of the metabolites included. This resulted in a total 194 out of 205 CAS numbers mapped to 298 ChEBI IDs, of which 48 mapped to a total of 133 WikiPathways.

**FIGURE 4:**
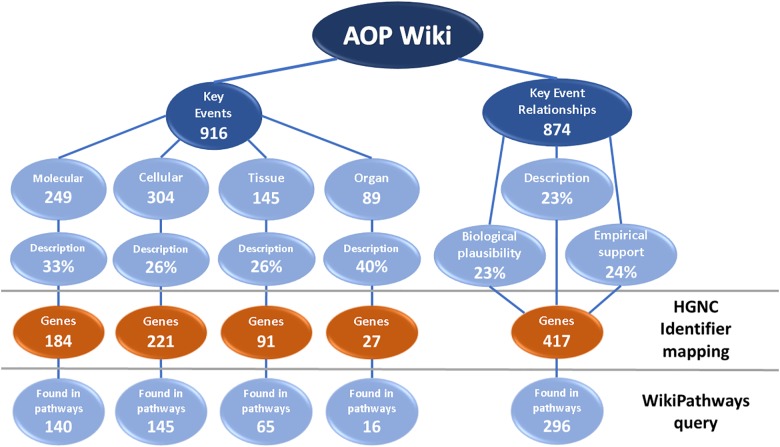
AOP-Wiki statistics on KEs and KERs, identifier mapping with HGNC identifiers and links to molecular pathways in WikiPathways. The KEs on the molecular, cellular, tissue, and organ level of biological organization and the KERs that connect them were parsed for texts of descriptions, on the biological plausibility and on the empirical support. HGNC Identifier mapping was performed to find all human genes described in the key event descriptions, after which these genes were queried on WikiPathways to find pathways that contain these genes.

As opposed to the hard linkage of the two databases, we also investigated a soft linkage, which entails the indirect linking of these databases through a text-based identifier mapping approach of human genes and performed a similar SPARQL query as for the metabolites (Figure 4). After extracting all KE descriptions from the AOP-Wiki, we mapped gene identifiers, symbols, alternative names, and previous names from HGNC to each description, leading to the identification of 523 genes in a total of 234 KE descriptions out of 787 KEs. In total, 70% of these genes were found in the molecular pathways of WikiPathways. Also, identifier mapping was performed on all 874 KERs that connect the KEs on the molecular, cellular, tissue and organ level. This was done on all texts for KER descriptions, biological plausibility, and empirical support, when available, and resulted in the identification of 417 genes, of which 296 are present in pathways on WikiPathways, which is 71%.

Furthermore, to benchmark the hard and soft connections between the AOP-Wiki and WikiPathways through ontologies and identifiers, we performed a full-scale manual check for all KEs on the molecular, cellular, tissue, and organ level of biological organization. This showed us that at least 2/3rd of all KEs can be mapped to molecular pathways on WikiPathways.

## Discussion

In this paper we explored possibilities for the integration of WikiPathways in the AOP-KB through ontologies, identifiers and manual judgment, to support AOPs and become a valuable tool in regulatory risk assessment. We looked at hard and soft linkages between the AOP-Wiki, the most actively used AOP module of the AOP-KB, and WikiPathways. We did this by extracting different types of information from the AOP-Wiki, such as chemical CAS numbers, KE and KER descriptions, and ontology annotations, and we performed a manual judgment of the linkage.

We found that the AOP-Wiki uses various ontologies to describe the different elements of KEs. To link the underlying molecular pathways to these KEs, we are mainly interested in the Biological Process that is annotated in the KEs, which describe the biology of the KEs. However, the ontologies currently used in the AOP-Wiki do not directly connect with the ontologies that describe the molecular pathways of WikiPathways. Consequently, manual effort is currently required to make this mapping, which negatively impacts the scalability.

Furthermore, we focused on the metabolites and genes/proteins described on the AOP-Wiki. For the metabolites, we parsed all CAS numbers, mapped these to ChEBI identifiers, and found that only 16% of these are found in WikiPathways. This is not unexpected, because most toxicological effects are caused by exogenous compounds, whereas WikiPathways mostly stores biological pathways containing endogenous metabolites. In fact, most WikiPathways that contain such a stressor do so because the pathway described the biotransformation of the toxic compound.

On the other hand, gene/protein identifiers that we obtained through mapping with an HGNC dataset did show high coverage by WikiPathways (70%). However, with the gene/protein identifier mapping, we only focused on human variants, although KE descriptions on the AOP-Wiki cover a variety of species. The taxonomic information is absent in most KEs and if it is available, the taxonomy identifiers are inconsistent, so we were not able to take this into account in our experiment of identifier mapping. Although species specification with ontologies does exist on the AOP-Wiki, the number of annotations and the consistency in reporting should increase for it to become a useful piece of data.

Apart from the automated linkages, we performed a manual check, which indicated that the majority of the processes in the AOP-Wiki KEs are covered by the WikiPathways database, either completely, as a part of a pathway or, in case of molecular interactions, the target molecule is part of a molecular pathway. This indicates us that there is potential in the interoperability of AOP-Wiki and WikiPathways to describe KEs. However, there is no one-to-one mapping of biological pathways possible. For example, molecular-level KEs currently often describe a single interaction between a list of stressors and a molecule, which would only be a part of a biological pathway on WikiPathways, besides the downstream cascade of molecular effects. Also, KEs on the tissue- and organ-level of biological organization are often non-specific. This could lead to the mapping of multiple molecular pathways to a single AOP-Wiki KE, even with the current WikiPathways content.

Besides the identification of connections between the AOP-Wiki and WikiPathways for improved descriptions of KEs, we aim for the possibility to introduce omics data analysis in the concept of AOPs. However, one concern mentioned in literature in the implementation of transcriptomics data in the concept of AOPs is the difference in the *causal* and *reactive* pathways (Sturla et al., 2014). Transcriptomics studies, for example, do not differentiate in its measurements between these two types of pathways, and by focusing on gene expression fold changes, pathway enrichment may highlight the reactive pathways. However, KEs may describe a causal event or pathway. Therefore, AOP-Wiki KE descriptions would not necessarily overlap with the results from pathway analysis with omics data. This should be taken into account in the descriptions of the molecular responses of KEs as this might impact the usability of omics approaches and their connections to KEs on the AOP-Wiki.

It is expected that omics approaches have great potential in the field of regulatory toxicology (Brockmeier et al., 2017; Buesen et al., 2017). However, there is a demand for well-described protocols and tools for omics data analysis and interpretation. The integration of WikiPathways in the AOP-KB as a data source and as omics data analysis tool allows more detailed descriptions of KEs and consistency in analysis and interpretation of omics data in the concept of AOPs. For that, you would ideally have molecular mechanistic descriptions for all AOP events in WikiPathways. The current analysis shows that useful connections already exist. To prepare for the integration of molecular pathways in the concept of AOPs, we created an AOP Portal on WikiPathways ^7^, in which all molecular pathways that are linked to AOP-Wiki KEs will be gathered and stored. This portal is meant to bridge the molecular knowledge and expertise of biologists and toxicologists to the framework of AOPs and allows the whole community to contribute to the collection of molecular pathways. This collection will be available for pathway analysis and network analysis with omics data for large-scale hypothesis generation for AOPs in response to a stressor or for biological read-across on the AOP level (Brockmeier et al., 2017). That would allow a more consistent, standardized approach for the integration of omics approaches in AOPs, and thus for regulatory use.

A variety of molecular pathway databases could fill this role as an omics analysis and interpretation tool for toxicological effects, such as KEGG and Reactome. However, molecular pathways can vary across pathway databases due to differences in pathway annotations by focusing on specific cellular contexts, such as diseases or specific cell types (Herwig et al., 2016). Moreover, Reactome and KEGG cannot be tailored like WikiPathways for specific communities or purposes such as described in this paper (Pico et al., 2008; Hanumappa et al., 2013). Besides, the accessibility of WikiPathways, being a community-driven, free-to-use molecular pathway database, fits with the existing AOP-KB modules and meets the requirements identified by the OECD: open access, standardized representation of data, and consistency in reporting (Pilat and Fukasaku, 2007; Ives et al., 2017). Because the AOP-KB is driven by a scientific community to develop, share and discuss AOPs, this community can also describe the molecular processes underlying the AOPs and contribute to WikiPathways and expand the AOP Portal.

Other work on the linkage of data related to the AOP-Wiki is the development of the AOP-DataBase (AOP-DB) (Pittman et al., 2018). This database will soon be publicly available and will contain various types of information linked to gene IDs that is useful for AOPs to provide a standardized, systematic structure for AOP development. Among a large amount of data, biological pathways from databases such as KEGG, Reactome, and ConsensusDB are included based on GO annotations of KEs in AOP-Wiki (Pittman et al., 2018). While the AOP-DB connects pathway databases based on the ontology annotations to of existing AOPs and assisting the identification of putative AOPs, we think that a direct link between KEs and molecular pathways would be valuable and more reliable.

In order to make a connection between AOP-Wiki and WikiPathways, we recommend a couple of improvements in terms of annotations and accessibility of the data. Since January 2018, the AOP-Wiki made available full XML files containing all data, which are stored as permanent downloads, as well as nightly exports of the full database. These files need to be parsed to retrieve the data, as described in this paper. This could be improved by developing an RDF version of the AOP-Wiki, allowing federated SPARQL queries to request all data, enable automatic information sharing, and has the use of ontologies as a core feature.

Furthermore, the current implementation of annotations with ontologies could be improved by annotating more specific elements of the KEs, as the existing KE components describe the KEs in general. More detailed annotations could be performed for many elements. For example, key genes, proteins, and metabolites should be annotated, as well as detection methods and biological assays, which can be annotated with ontologies such as the Chemical Methods Ontology or BioAssay Ontology. Also, when biological pathways are described in a KE, annotations with the Pathway Ontology would allow a direct connection to the WikiPathways database including all genes, proteins, and metabolites involved, which are annotated with various databases through BridgeDb in the WikiPathways diagrams.

Besides the ontology annotations, the only molecules annotated on the AOP-Wiki are the chemicals related to stressors, which are identified with CAS numbers. However, not all of these CAS numbers are linked to open structure data that is incorporated in the BridgeDb mapping that we performed. It is essential that these CAS numbers are included in public databases, such as WikiData (Mietchen et al., 2015) or that public database identifiers are used, such as from ChEBI or even Wikidata as an outside database for chemical information. Besides chemicals, nanomaterials, which are extensively investigated for toxicity, also require annotations, for example with the eNanoMapper ontology (Hastings et al., 2015). Also, the free-text descriptions of KEs that describe the biological process can also be improved by more consistent reporting, such as a fixed vocabulary for all genes, proteins, and metabolites involved in the biological processes. For example, listing the most important molecules by HGNC symbols or ChEBI IDs for a KE would improve machine-readability and the automated discovery of new connections between KEs.

On the other hand, WikiPathways will also need to undergo updates to fit the connection as described, with a specific category of KE-related molecular pathways and the need for so-called meta-pathways to create an AOP Network. Also, the AOP Portal will be populated with pathways in a case-study approach, proving the usefulness of the database. Other improvements related to toxicity research is the linkage to kinetics databases, more info on post-translational modifications of proteins, and improved semantic annotations of localizations, for example, specific organelles, cells, or tissues.

Taken together, we claim that a tight integration of WikiPathways and AOP-KB will improve risk assessment because we can link omics data directly to KEs and therefore AOPs. However, to make assessment reproducible and valid, major changes are needed.

## Data Availability Statement

The AOP-Wiki dataset analyzed for this study can be found in the AOP-Wiki (aopwiki.org/downloads). The WikiPathways data used for this study can be found in the WikiPathways database (data.wikipathways.org).

## Author Contributions

MM and EW designed the study and did the analyses. TV performed the manual matching of the databases. MM wrote the first draft of the manuscript. LB and HA wrote sections of the manuscript. PN, FA, EW, and MM contributed to pathways in the WikiPathways AOP Portal. All authors conceptualized the study, revised the drafts, gave the feedback, and approved the final manuscript.

## Conflict of Interest Statement

The authors declare that the research was conducted in the absence of any commercial or financial relationships that could be construed as a potential conflict of interest.

## Footnotes

1 https://aopkb.oecd.org/index.html

2 https://aopwiki.org/

3 https://effectopedia.org

4 http://wikipathways.org

5 https://aopwiki.org/downloads

6 http://sparql.wikipathways.org/

7 http://aop.wikipathways.org
